# Wernicke’s Encephalopathy Presenting as Sensorineural Hearing Loss

**DOI:** 10.7759/cureus.7378

**Published:** 2020-03-23

**Authors:** Benjamin S.H. Bryant, Paul D Ehrlichman, David Hewson, John W Sanders, Christopher H Chu

**Affiliations:** 1 Internal Medicine, Wake Forest Baptist Medical Center, Winston-Salem, USA; 2 Infectious Diseases, Wake Forest Baptist Medical Center, Winston-Salem, USA

**Keywords:** wernicke, encephalopathy, hearing loss, thiamine

## Abstract

Sleeve gastrectomy is a restrictive-type weight loss surgery that can result in nutritional deficiencies, such as vitamin B1 (thiamine) deficiency. Although Wernicke’s encephalopathy is a known complication following bariatric surgery, bilateral sensorineural hearing loss is a rare presenting symptom of this condition. We present a case of a patient two months postoperative following a sleeve gastrectomy whose chief complaint was hearing loss. While initial laboratory evaluation of her complaint showed elevated inflammatory markers, prompting an autoimmune workup, ultimately the diagnosis of Wernicke’s encephalopathy was confirmed by a low thiamine level and magnetic resonance imaging findings. A correction of the patient’s thiamine deficiency led to an improvement of her symptoms.

## Introduction

Laparoscopic sleeve gastrectomy (LSG) is a restrictive-type weight loss surgery that has gained favor in comparison to other bariatric procedures for its lower complication rates [1]. LSG is associated with fewer long-term nutritional deficiencies when compared to other bariatric procedures. The presumptive difference is that intestinal surface area is unaffected and therefore intestinal absorption remains intact. Nevertheless, there remains a subset of patients who have vitamin B1 (thiamine) deficiency after their procedure, putting them at risk for developing Wernicke’s encephalopathy (WE). Risk-factors that put LSG patients at increased risk of thiamine deficiency include nausea, vomiting, African American race, and greater preoperative body mass index (BMI) [2]. Other risk-factors have also been identified, such as noncompliance with vitamin supplementation, poor diet, decreased food intake, and prolonged parenteral feeding [3, 4]. Hearing loss associated with WE is rare, and has only been documented in some case reports [5]. Even though the incidence of bilateral sensorineural hearing loss (SNHL) in patients with WE is unknown, it has often been associated with WE cases unrelated to alcohol consumption, particularly in young women [6]. We present a report of a patient presenting with hearing loss two months postoperative following a sleeve gastrectomy; audiometry results were consistent with bilateral SNHL. She was ultimately determined to have WE due to poor oral intake as indicated by a low thiamine level and suggestive magnetic resonance imaging (MRI) findings. The aim of this paper is to draw attention to bilateral SNHL as a rare presenting symptom of WE, especially in the context of bariatric surgeries in young females without a history of alcoholism.

## Case presentation

**Figure 1:**
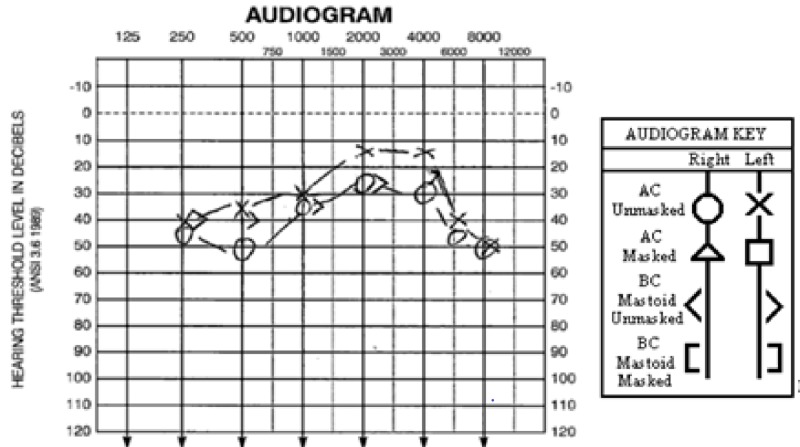
Audiogram results showed moderate, bilateral reverse trough sensorineural hearing loss. The audiogram key is provided indicating air versus bone conduction, and right versus left ear. AC: Air conduction; BC: Bone conduction.

The patient is a 22-year-old African American female who presented to the emergency room with two months of decreased oral intake, 10 days of bilateral lower extremity weakness, and hearing loss. The patient had undergone sleeve gastrectomy two months prior at another institution. In the postoperative period, she had presented twice to the same outside institution for recurrent esophageal strictures causing episodes of decreased oral intake. These strictures were often relieved by esophagogastroduodenoscopy (EGD) with dilatation. Our patient had recently been evaluated at this institution a few days prior for worsening of her symptoms, with no diagnosis given or resolution of her symptoms achieved. Upon presentation to our institution on Day 1 of hospitalization, she was alert and oriented to person and place only, drowsy, and had 3/5 strength of the bilateral lower extremities. Initial laboratory workup demonstrated an anion gap metabolic acidosis with a positive beta hydroxybutyrate and ketones seen by urinalysis, all in the setting of poor oral intake suggestive of starvation ketosis. A thiamine level was drawn at that time. The patient denied any consumption of alcohol in the months since her LSG. On Day 2, an EGD was performed which showed no evidence of stricture. At that time, the patient was started on a pureed diet with proper vitamin and nutrient supplementation. Audiometric testing was also performed that morning, and showed moderate, bilateral reverse trough SNHL (Figure 1).

**Figure 2:**
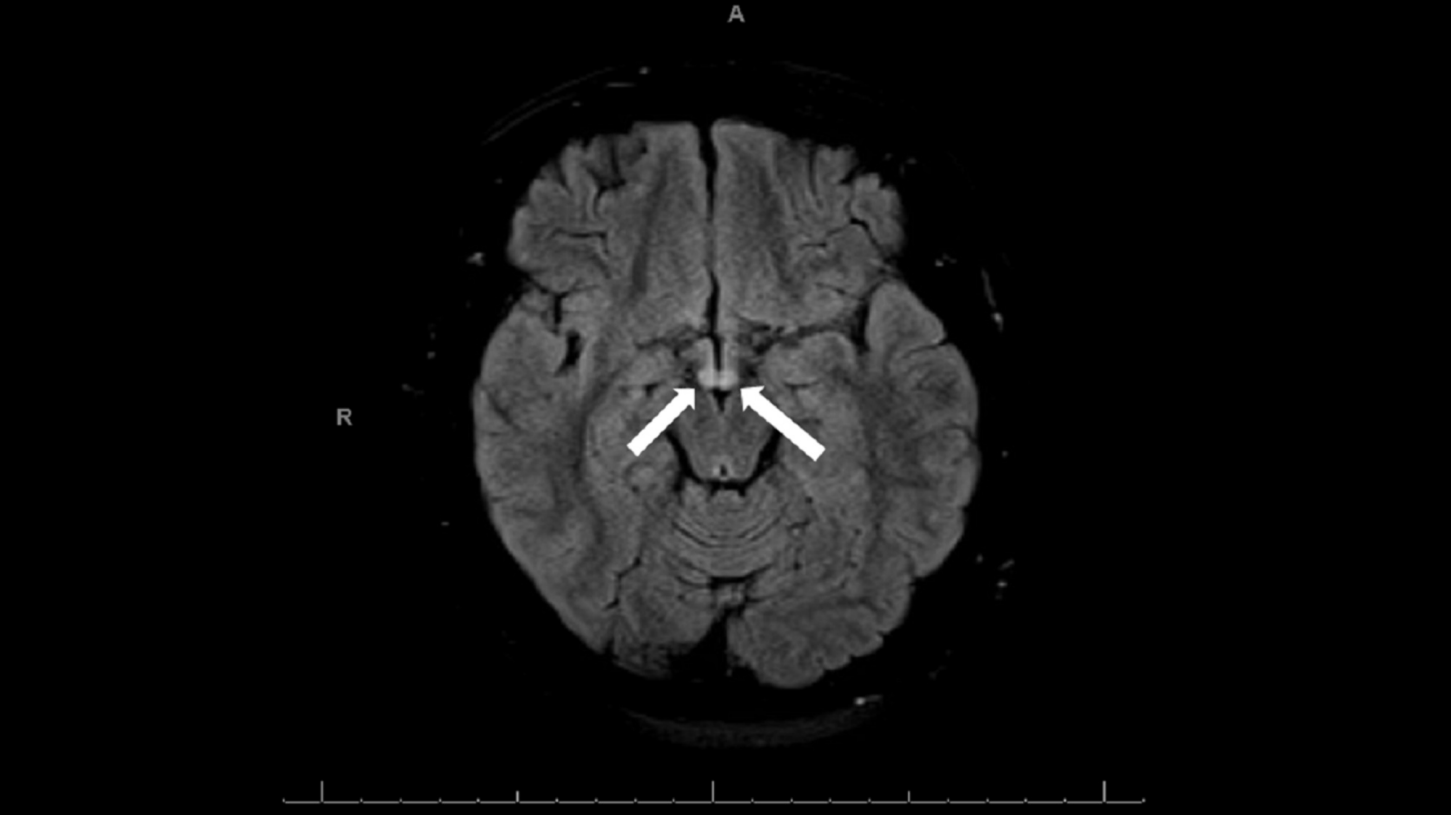
MRI of the brain with and without contrast showed increased signal on T2 FLAIR imaging in the mammillary bodies bilaterally, and enlargement of both mammillary bodies. The radiologist's interpretation was that these findings can be seen in Wernicke’s encephalopathy. FLAIR: Fluid-attenuated inversion recovery; MRI: Magnetic resonance imaging.

Given the abnormal audiometry results, the otolaryngology team was consulted on Day 3, with recommendations to begin empiric steroid therapy and assess for an autoimmune etiology for the findings. Additional labs revealed an elevated C-reactive protein (44.9 mg/L), elevated erythrocyte sedimentation rate (83 mm/hr), and a positive rheumatoid factor. On Day 4, MRI of the brain was ordered, with findings showing bilateral mammillary body edema and enlargement (Figure 2).

**Figure 3:**
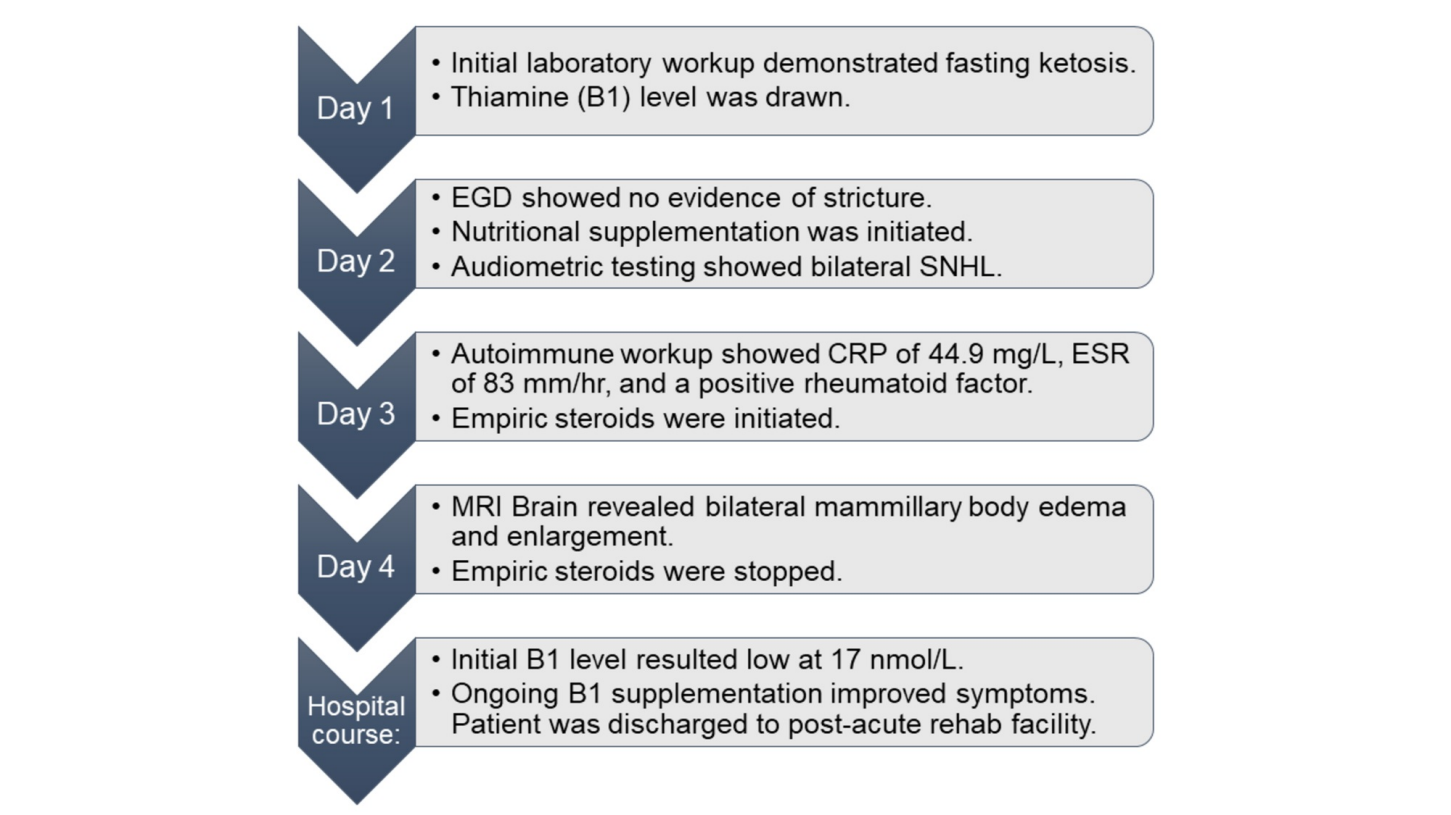
Hospitalization timeline, including the pertinent diagnostic and therapeutic events. CRP: C-reactive protein; EGD: Esophagogastroduodenoscopy; ESR: Erythrocyte sedimentation rate; MRI: Magnetic resonance imaging; SNHL: Sensorineural hearing loss.

Given these findings, empiric steroid therapy was discontinued. The patient's thiamine level resulted on Day 5, and was low at 17 nmol/L (70-180). Following correction of the patient’s thiamine level, the patient exhibited gradual improvement in her symptoms. At the time of discharge (Day 11), her hearing loss and lower extremity weakness had nearly resolved. She was discharged on B1 supplementation with plans for post-acute short-term rehabilitation and physical therapy. Upon completion of rehabilitation, her strength and stamina improved, and she was able to return home with her family. She continued to follow-up with our institution’s neurology department, as well as our institution’s bariatric surgery department. A repeat thiamine level was checked three weeks after her discharge date, and was found to be in normal range at 128 nmol/L. A summary of this patient’s hospitalization with a timeline is shown below (Figure 3).

## Discussion

WE is a known complication following bariatric surgery, with risk factors for WE depending on the type of bariatric surgery performed [7]. Procedures such as duodenal switch or Roux-en-Y gastric bypass have increased risk of nutritional deficiency secondary to alterations of the gastrointestinal tract structure. LSG, however, only involves removal of a portion of the stomach, with its ultimate aim to decrease volume of food consumption and lower secretion of the hormone ghrelin that regulates hunger. Even though intestinal absorption remains intact after an LSG, thiamine deficiency can still occur in these patients [4]. Multiple studies have documented thiamine deficiency and WE after Roux-en-Y gastric bypass, but the literature investigating the incidence of WE after LSG has been limited to case reports [2]. It has been reported that 25% of LSG patients have low serum thiamine levels within two years of operation, and up to 30% of them within five years [8]. Risk-factors that put LSG patients at increased risk of thiamine deficiency include nausea, vomiting, African American race, and greater preoperative BMI [2]. Other risk-factors have also been considered, such as noncompliance with vitamin supplementation, poor diet, decreased food intake, and prolonged parenteral feeding. When WE is not adequately treated it has a 17% mortality rate, or can lead to Korsakoff’s syndrome, a serious neurological complication characterized by chronic memory dysfunction [9]. Unfortunately, given the variability of patient presentation, as well as the fact that thiamine levels can take days to result, the diagnosis of WE is often missed in its early stages [10]. In fact, a case series of post-mortem autopsies found that the diagnosis was missed in about 75-80% of patients [11].

Upon literature review, it is clear that hearing loss associated with WE is a rare presenting symptom with an unknown incidence. The mechanism of its pathogenesis is thought to be due to involvement of the ascending afferent auditory fibers in the brainstem [5]. A previous case report performed a review of English literature revealed eight cases of hearing loss associated with WE [6]. The hearing loss seen was often bilateral in nature and seen in young women aged 17-35 years who did not have any history of alcohol abuse. Three of these cases were associated with a history of bariatric surgery. None of the reviewed cases reported audiometry findings. The case presented in our report is distinct yet consistent with such findings, in that we present a case of a 22-year-old female who was two months postoperative following a sleeve gastrectomy whose chief complaint was bilateral hearing loss, with audiometry findings providing objective data.

In our patient, elevated inflammatory markers led us to consider an autoimmune etiology for her hearing loss. However, these laboratory findings are overall nonspecific, and do not necessarily suggest an autoimmune pathology. In the diagnosis of WE, biomarkers such as a plasma thiamine level do not correlate with the diagnosis of WE [12]. While MRI findings are considered the most helpful diagnostic modality (with a specificity of 93%), the sensitivity of these findings is only 53%. Only about 60% of patients with WE after LSG will have the typical MRI findings [13]. Diagnostic testing should not delay the initiation of treatment with thiamine. Given the morbidity associated with the neurological sequalae of WE, as well as the fact that the neurodegeneration associated with WE can only be reversed with early thiamine treatment, there should be a high index of suspicion for WE in at-risk populations that present with neurologic complaints [14].

## Conclusions

As bariatric procedures such as LSG gain favor in the United States’ efforts to lower morbid obesity rates, it is important to be mindful of long-term complications such as nutritional deficiencies. Thiamine deficiency and WE rates have increased in proportion to the relative increase in the number of gastric bypass surgeries being performed in the US. The presenting symptoms of WE are often atypical. Given this variability, as well as the fact that thiamine levels can take days to result, the diagnosis is often missed in its early stages. Given the morbidity associated with the neurological sequelae of WE, there should be a high index of suspicion for WE in at-risk populations that present with neurologic complaints.
